# Experiences of Parents with Opioid Use Disorder during Their Attempts to Seek Treatment: A Qualitative Analysis

**DOI:** 10.3390/ijerph192416660

**Published:** 2022-12-11

**Authors:** Christine Bakos-Block, Angela J. Nash, A. Sarah Cohen, Tiffany Champagne-Langabeer

**Affiliations:** 1 Center for Health Systems Analytics, School of Biomedical Informatics, UTHealth Houston, Houston, TX 77030, USA; 2 Cizik School of Nursing, UTHealth Houston, Houston, TX 77030, USA

**Keywords:** opioid use disorder, parents, barriers, gender, stigma

## Abstract

In the U.S., 12.3% of children live with at least one parent who has a substance use disorder. Prior research has shown that men are more likely to seek treatment than women and that the barriers are different; however, there is limited research focusing specifically on opioid use disorder (OUD). We sought to understand the barriers and motivators for parents with OUD. We conducted a qualitative study by interviewing parents with OUD who were part of an outpatient treatment program. Interviews followed a semi-structured format with questions on access to and motivation for treatment. The interviews were recorded and transcribed using OpenAI software. Transcripts were coded by two separate reviewers and then analyzed for themes using Atlas.ti. We interviewed 14 individuals; 3 were men, and 3 of the women identified as LGBTQ+. The participants ranged in age from 27 to 54 years old. All participants had a least one child. Gender differences existed. Mothers reported experiencing more barriers—notably, a lack of childcare, shame, and guilt—while fathers reported higher levels of support from family. Both mothers and fathers identified their children as a motivation for recovery, albeit in differing ways. Mothers and fathers with OUD experience different barriers to treatment and also rely on different resources. Prior efforts to increase access to treatment for parents have focused on physical barriers; however, our research supports the need for expanded treatment services for families and efforts to address the stigma of substance abuse disorder, but more efforts are also needed to address stigma.

## 1. Introduction

Approximately 8.7 million children in the United States live with at least one parent who has a substance use disorder (SUD) [1]. Of those children, 7 million live in a two-parent household, and 1.7 million live in a single-parent household [1]. It is estimated that the majority (1.4 million) of these children live with single mothers, and approximately 344,000 live with single fathers [1]. Parental substance use is a major contributing factor to the rise in child welfare cases nationwide, contributing to 26% to 34% of all cases between 1990 and 2016 [2]. Consequently, the children of individuals with an SUD are more likely to have a lower socioeconomic status, experience problems in academic and social development, and are more likely to use illicit substances in adolescence [1,3,4]. Despite these impacts, access to treatment options for SUDs remains challenging for parents and particularly for mothers [5,6].

Historical data indicate that men may be more likely to seek treatment for an SUD than women, and ongoing efforts have stressed the need for dedicated treatment for women [6,7]. The U.S. Department of Health and Human Services, Office of Women’s Health recommends that treatment programs work to eliminate gender disparities in substance use treatment and research, but few programs offer services tailored to the specific needs of women [8,9]. Although less research has focused specifically on parents with opioid use disorder (OUD), women are more likely to suffer from chronic pain as well as co-occurring mental disorders [2,10,11]. Women may also experience more intense withdrawal symptoms than men, requiring a close dosage monitoring of medications for OUD due to fluctuating hormonal interactions [12,13,14]. Compared to men, women are more likely to have a greater history of adverse childhood experiences and physical health problems, which can complicate the recovery process [15,16,17,18]. Many individuals with OUD are parents and face increased barriers to accessing treatment. There has been a great deal of research focused on the quantitative variables which contribute to the success or failure of treatment; however, we sought to identify the barriers experienced by both mothers and fathers using individual interviews and qualitative methodologies. This study seeks to understand, from a first-hand perspective, the impact of a parent’s opioid use and subsequent recovery on their parenting and family systems and to discover the sources of support that mothers and fathers need to help their children during active use, treatment, and recovery.

## 2. Materials and Methods

### 2.1. Study Participants

The participants were self-selected from a weekly peer support group for patients with opioid use disorder. During peer support groups from September 2021 to January 2022, peer support specialists announced the research project and provided contact information for the investigator for anyone interested in participating. Respondents were reviewed for eligibility based on the following criteria. The inclusion criteria were being at least 18 years of age, having minor children at the time of addiction and entry into treatment, and being currently in treatment. Qualitative research methods were selected for this study to provide an in-depth exploration of the subjective experiences of the parents with OUD. The techniques used in qualitative inquiry are significantly dissimilar to those that are necessary when trying to calculate the prevalence, distribution, or numerical differences of a particular group. The goal of external validity in qualitative inquiry is to determine if the study’s hypothesis and results can be applied to other contexts. To do this, contextual background information, including descriptive characteristics of the sample and the environment, is included. Additionally, the findings are not supposed to be valid for population groups at large but for other individuals in similar contexts. Random sampling is therefore rarely a relevant tool for validity in these studies [19]. We sought to understand their individual experiences, prior to recovery, during their attempts to seek treatment, during treatment, and in the different stages of their recovery process. The protocol for this study was approved by the university’s Institutional Review Board, and informed consent with permission to record was obtained from each participant prior to data collection. Participants received a USD 10 Walmart gift card for their time.

### 2.2. Data Collection

The participants were individually interviewed by either a primary female with a Doctor of Philosophy (PhD) in social work and a Licensed Clinical Social Worker (LCSW) or a backup female PhD in clinical nursing with advanced training in mental health. The interviews took place via videoconference between 16 September 2021 and 14 January 2022. The interviews were semi-structured and used a set of questions as a guide (see Supplement S1). Interviewers were not required to ask all questions, and questions could be asked in any order. Additionally, interviewers were allowed to ask follow-up questions with the intention of keeping a normal flow to the conversation. All interviews were recorded and transcribed using open-source speech-to-text translation software (OpenAi Software, Temi.com).

### 2.3. Analysis

The primary interviewer checked the transcriptions of all interview recordings for accuracy. The transcriptions were then manually redacted to remove identifying information. Once the redaction was complete, the initial transcriptions and recordings were deleted to protect the privacy of the participants. The transcriptions were analyzed in Atlas.ti (Version 22). First-level coding was conducted by two members of the research team, who each coded the same three interviews and met to compare, discuss, and resolve differences in coding. The remaining interviews were then divided between the coders, who continued to meet to ensure internal consistency. Finally, to increase the trustworthiness of the data, a third member of the research team reviewed the completed codes for validity. Finally, content analysis was used to identify themes and subthemes related to accessing treatment and the recovery process among the mothers and fathers enrolled.

## 3. Results

### 3.1. Participant Characteristics

Fourteen individuals were interviewed for this research. All participants had minor children during their addiction and initial entry into treatment. Table 1 provides a demographic summary of each of the participants. Three females identified as lesbian, gay, bisexual, transgender, or queer (LGBTQ+). Participants ranged in age from 27 to 54 years and had stable housing at the time of the interviews. There were three main themes that emerged from the interviews: barriers to treatment, resources, and motivation. Common words from the transcript include “kids” (or a variation such as “son”/“daughter”/“child”), “support”, “family”, “help”, “stigma”, and shame.

### 3.2. Theme 1: Significant Barriers to Accessing Care

All of the fourteen participants expressed that they had experienced significant barriers to accessing treatment for their opioid use disorder. Among the barriers to accessing care, the reasons provided were having custody of children (n = 11, all mothers), stigma and discrimination (n = 10), a lack of knowledge (n = 5), the availability of care/distance (n = 3), having a criminal record (n = 2), and cost (n = 2). Only mothers said that having children in their care prevented them from entering a treatment facility. One mother discussed her involvement and fear as she interacted with Family and Protective Services: “There were so many times they told me to get treatment, or you can lose your kids. But they don’t tell you where to go with your kids, or what to do with your kids when you are in treatment” (Female 4). Others remarked: “They told me to get treatment, but I didn’t have anywhere for my children to go. Their dad wasn’t around and no real family to help after my mom died” (Female 8). One mother commented on her challenges in a single-parent household: “It was really hard for me to find a treatment that I could take my child with me, because I didn’t have anywhere for my child to go for me to go to treatment” (Female 10).

Gender differences in the way people with addiction are perceived were also felt by the mothers we interviewed. Several commented on the perceived difficulty that mothers faced over fathers. One mother said: “I don’t think it’s the same with fathers, because we carry the babies, you know? Like, when people look at moms, like how, how could you do that when you’re pregnant?” (Female 9). A second mother discussed the stigma that mothers faced: “Oh, I think moms in recovery have it way worse than dads. We bear the stigma. We have more to prove. With dads, people are like, ‘oh, he’s doing so good’ and with moms, people are like, ‘how could she do that to her kid?’” (Female 1). Three of the mothers identified as LGBTQ+ and reported feeling stigmatized because of their sexuality. One mother who identified as gay stated: “I was kind of double-whammied because I’m gay and then I had a child, and I’m a single mom that’s gay, and then add the stigma” (Female 10).

The stigma felt by the participants of the study was extended to the healthcare setting. When discussing mistreatment by healthcare and medical workers, mothers were far more outspoken than the fathers in our study. One mother noted: “I can’t stand the doctors. Because I feel judged by them immediately, anybody with any kind of medical education, you can take one look at my skin and know that I’ve been a drug addict. And yeah, I definitely feel judged immediately when I go to places like that” (Female 4). Another talked about her experience with the hospital: “I had gone into a hospital and tried to seek help, you know, I didn’t know what was wrong. I ended up finding out I was pregnant and I had track marks, I’m on drugs, I got all these things in my system and instead of helping me find resources, like, um, a nurse or a social worker that is in the hospital to help get off the drugs, or do something to help that part of the addiction, they just sent someone in there to see if I wanted to give my baby up for adoption” (Female 11). Another mom said: “I have so many scars on my body from staph infections, and from being an IV drug user. And I feel like when I go to the ER for any reason, and even when I’m clean, I still feel this way. I feel stereotyped immediately. I feel I don’t get the same treatment as other patients” (Female 4).

Seven of the participants, who were all mothers, reported a lack of knowledge about where to get services, the cost for services, or how to access services as a barrier to treatment. One participant explained how she felt lost in the healthcare system: “They told me ‘You need to get help’ but they didn’t tell me where I could get help or how I could get help. They just said I needed to get help. And here I am, and I’m trapped in an opioid addiction and there’s no solution for me. I don’t know a way out. All I know is CPS is trying to take the only good thing I have, which is my child from me. And I can’t stop using. Um, and I don’t know what to do” (Female 10). Further, several respondents noted an inability to pay for treatment services: “Did they not notice that my bills exceeded my income? Like I have 20 bucks left over after I pay my bills. You know, and they really expect me to pay $83 to see the doctor? I was shocked” (Female 4). One remarked: “Before I had a job and I took meds I had to get assistance to pay for them and fill out these forms. The suboxone was really expensive, like over 100 bucks a month. And when I got a job I had to pay for them myself” (Female 5).

Interestingly, the fathers we interviewed reported barriers but did not report the same difficulties faced regarding children when compared with the mothers in the study. One father discussed his criminal record as an obstacle to recovery. He explained that most people he knew had criminal records; however, once they were in recovery, it was difficult for them to move forward, to find stable housing, and to seek employment: “It’s hard for me to get a job because you know, I have so many felonies” (Male 3). It is also worth noting that none of the fathers in our study had custody of their children. More significantly, fathers discussed treatment availability and distance as barriers, one dad commenting: “I guess at one point there was like, there was everywhere, the beds were full everywhere. But that was only for a week. I mean, the only think that would make it hard for someone to find treatment is if they’re making excuses and don’t want to go yet” (Male 2).

### 3.3. Theme 2: Resources Are Needed by Participants

Participants recalled both resources they had and resources they wish they had in order to access treatment. Seven participants (50%) stated that coaches and counselors were essential, while family (n = 5), friends (n = 3), and religion/church/faith (n = 2) were also listed as strong supports for recovery-seeking behaviors. One mother expressed her relationship with a counselor in this way: “After they took my daughter, I was arrested for possession of a controlled substance. I was previously on probation, so I was arrested and held with no bond. I expressed all these things to the judge and that was my pivot point of recovery. He put me in treatment and as soon as I got to treatment, I got with my counselor, and I got my service plan and started getting into action right away” (Female 6). Dads reported the support they had from counselors, as well as family. One dad said: “I love talking to [redacted], which is my counselor, every week, being able to express how I’m doing, what’s going on with me. And it’s helped a lot” (Male 3). The mothers also discussed the support they received from counselors, others in the recovery community, namely, other women. One noted: “I loved talking to my counselor and being able to talk about what I was going through” (Female 9). Another mother said: “I get support from like the, like the anonymous groups in like, uh, at my clinic. I have a counselor, my methadone clinic, a counselor that is really, like she’s really helped me out a lot as far as getting sober and she’s kind of pushed me along” (Female 3). Many participants explained that the instruction and feedback they received from coaches and counselors were similar to a safety net or surrogate family, especially in cases where their own biological family members were not available to provide support.

Mothers and fathers, however, seemed to have different levels of support from their family. Fathers in our sample tended to rely on the mothers of their children or their own mothers for support. One father remarked: “He’s got a good mom, so she really took care of everything… We’re (his child’s mom and him) still cool, always been because her dad was an alcoholic, and so she knew what recovery was like” (Male 1). Another father said: “I was very well covered with family support, for sure” (Male 2). The fathers were also overwhelmingly positive when discussing their family and social support: “I’m really blessed and on the lucky side of it, I have, you know, a lot of support. Recovery capital is what they call it” (Male 2). Another father stated: “My mom has always been supportive. My grandmother, before she died was supportive. Even my son’s mom was supportive” (Male 1). Contrary to fathers, mothers seemed to lack support and discussed the struggle they felt to navigate treatment options alone. Several lamented not having a friend or family member to help them. This was due to multiple reasons: not living near parents or family members, family members also living in patterns of addiction, or disagreements with family members. One mother commented: “Um, honestly, I didn’t have support. That’s why I don’t have them (her children). I think if I would’ve had support, just, you know, somebody that cared or checked on me, things would have been different. I think I’d have custody of my kids now” (Female 2).

Faith in God was identified as an important resource for mothers. One mother described her faith in God: “It really made a powerful impact in my life, you know… with God and prayer, praying every day gave me time to heal” (Female 9). Another mother said: “It’s very important to have faith in God, a higher power, yourself, or whatever you believe in, that can help you push through on a bad day” (Female 6). Another mother described how faith in God was passed down to her from her mother: “My mother showed us, she’s the one that showed us about faith in a higher power, you know, the Lord Jesus. And so, I always knew it inside of my heart, that there was somebody there that did care and that it was him” (Female 8). Fathers in our study talked about turning to others when they needed additional support and learning to talk about their feelings. One father said of his family: “They help me with my day-to-day routine. We usually sit down over coffee, and just talk, I get to really express what’s just going on in my mind and how I feel… I think that’s the biggest thing for me, you know, to be open and honest about how I’m feeling” (Male 3). Another dad said that his girlfriend helps him when he needs it: “I got someone that tells me to go to meetings and encourages me to go to meetings. She understands that if, I don’t have that recovery in my life, that our relationship ain’t going to work” (Male 2).

### 3.4. Theme 3: Motivation to Seek Treatment

Participants had the most similar answers when asked about their motivation for seeking treatment. Eleven participants stated that children were an essential factor, and approximately a quarter felt that self-motivation was key (n = 4). Other reasons given included a fear of death (n = 3), and two of the fathers expressed a fear of going to jail. Although mothers and fathers differed in terms of their barriers to treatment, both equally stressed their children as a motivation. Their responses had slight nuances in the desired outcomes. For example, mothers tended to use their children as the motivation to seek care, while fathers stressed the importance of seeking care to reciprocate in a mutual relationship with their children. One mother said: “My children were like, my motivation. I made a commitment a long time ago. A lot of times people say ‘you gotta do it for yourself, you’ve gotta do it for yourself.’ And I did, I did it for myself, but I did it for my children too, I did it for them. And I’m doing it today for my children. I still do” (Female 9). Although these comments may seem contradictory, both parents stress the importance of committing to recovery for their children. One father said: “This is how I make things right again, by staying off drugs and going to meetings, Like I said, now, my son comes, and he’s even made the comment of ‘I got my dad back’” (Male 3).

Although many parents stated extrinsic factors, some parents felt that intrinsic or self-motivation factors were more important. One father commented: “Some think that it might be a motivation, you know, your kids and your family… I think that it can’t be a motivation really. It has to be you. You have to commit so much of your recovery and be selfish in a good way, that will benefit your kids in the future” (Male 2). He continued to discuss why self-motivation was more important than other factors: “I had so many failed attempts at recovery, from like I said, putting my son first or just saying, ‘oh I can’t do this, I don’t have time to make these meetings because I have to do this’, or whatever for my son. When you don’t put yourself first, you won’t be able to see your son anyway, he’ll be seeing you in a casket” (Male 2). One mother discussed a future life she would like to have—one where she did not use illicit substances: “I always dreamed of, you know, being someone and having things, that being happy and doing things, having a life. Being a mother to my children” (Female 5).

Many of the participants (n = 5) mentioned fear as a motivation for seeking treatment. Participants told stories of overdosing more than once and being brought back to consciousness with naloxone by first responders. One father said: “I flatlined for about 2 min, but they brought me back… They (his mother) called 9-1-1. Um, I went to the hospital, she called my probation officer and my therapist, asking if they would take me back, or you know, what the next step was… That’s what got me to where I am today... I have 1 year sober” (Male 3). Another father stated: ”I had my first overdose; I was dead for eight minutes” (Male 2). Similarly, when discussing what finally got them to treatment, both mothers and fathers stated that entering the criminal justice system was a fear, or entering into treatment was a consequence of entering the system. Fathers reported going to jail or prison as a general motivation for seeking treatment, one father stating simply: “They offered me eight months in state jail or six months in treatment. So, I didn’t want to go to jail, so I chose treatment” (Male 3). Another said: “It was my third time going to prison. It was getting old, going to prison” (Male 2). Mothers, however, mentioned avoiding negative consequences and jail as a motivation for entering treatment or requesting alternate arrangements or sentences. One mother, regarding the possibility of her using again, said that: “I could go to jail because it breaks my probation contract, because I’m in recovery” (Female 7). Fathers also discussed the negative stigma associated with finding work with a criminal background. One father said: “It’s hard for me to get a job, you know, I have so many felonies... it’s a vicious cycle” (Male 3). One mother asked a judge to send her to a long-term treatment in an outpatient facility, understanding that she needed a higher level of care to attain sobriety. This mother said: “I actually asked the judge to send me to [facility name], where they have a 2-year program, because I knew if I got out that I was going to use again. She sent me to a 9-month program, because of my children” (Female 9). Another mother stated: “They wanted to send me to state jail. I advocated to the judge that that will not help me and my daughter” (Female 6).

## 4. Discussion

This study explored the experiences of parents with opioid use disorder during their attempts to seek treatment. Although our study was small, the themes uncovered are supported by the literature. Individuals seeking help face significant barriers to accessing care. These are present in multiple forms, including stigma and shame, a lack of knowledge, and childrearing responsibilities. As the participants were all parents, the topic of children was discussed in multiple contexts. Our study found that mothers were more likely to enter treatment to become better caretakers in order to shield their children from their substance use and to achieve abstinence. This is consistent with other findings according to which mothers have entered treatment to maintain or regain custody after a child was removed from their care [20]. On balance, children were also seen as a barrier for mothers for entering treatment, as the majority of treatment centers mentioned by participants did not have childcare options. Mothers in our study spoke extensively about the difficulties in finding treatment that would accommodate their children. Existing research supports our findings that lacking childcare and the absence of child-friendly programs presents a barrier to parents seeking treatment and recovery support [21]. Residential and out-patient programs that are family-centric and include services for children would be beneficial in helping treatment-seeking parents. For mothers with children, this meant less of a capacity for seeking care. However, the fathers in our study and other studies were less likely to have physical custody of their children [22,23].

In addition to a lack of childcare options, there were other barriers that were disproportionately felt by mothers. The cost of treatment is a well-documented barrier, and two participants in our study mentioned the cost of treatment as a barrier. One commented on how becoming employed reduced her public benefits, making the cost of treatment prohibitive [5]. Medicaid is the largest payer of substance use treatment and covers over 40% of all births in the U.S. [24,25]. However, in states that did not expand Medicaid, women may lose their health coverage just 2 months postpartum [26]. This is especially problematic, as a mother’s likelihood of relapse with OUD peaks between 7 and 12 months postpartum, leaving many mothers with OUD with few to no affordable options for treatment [27].

Nearly USD 3 billion in costs associated with parental opioid use (about 2.1% of all child welfare costs) were incurred in the child welfare system between 2011 and 2016 [2]. Parental opioid use accounted for more than 200,000 reports of child abuse and neglect, over 80,000 of which were substantiated, and more than 95,000 children entered foster care. The presence of illicit drugs in the home not only increased child welfare cases but also increased the risk of accidental ingestion by children [28]. Indeed, pediatric opioid-related overdose has drastically increased over the last decade, with children 3 years and younger at an increased risk of accidental ingestion [29].

The results from our study also indicate that stigma and shame, particularly for mothers, represented barriers to seeking treatment. Women with OUD who have children face more stigma than those without children; further, mothers in our study and others reported a constant fear of judgement and losing custody of their children as a result of seeking treatment [5,30,31]. The prevalence of unintended pregnancy among women with OUD is exceedingly common, with over 85% reporting unintended pregnancy compared to 45% of women in the general population [32]. Often, pregnant women with OUD avoid prenatal care due to fears of losing custody of their children [33]. Some of these fears are based on policies that remove children from mothers in treatment, and other fears are the result of repeated, institutionalized stigma from family and healthcare professionals [34,35]. The stigma surrounding substance use leads to blaming a person for their choices. About one-third of people in treatment reported experiencing stigma from friends and family and being portrayed as “untrustworthy” and “irresponsible” [36]. A survey of primary care providers in the U.S. found that providers prefer not to prescribe medications for OUD because of the belief that patients are “disruptive, manipulative, and demanding” and may cause existing patients (who do not use drugs) to leave the practice [36]. Our study found that when individuals experienced discrimination and stigma from healthcare providers, they were less likely to seek treatment or preventive or acute care services.

Many in our study stated that they could not have entered treatment without the assistance of an outside source. The fathers in our study talked about their children’s mothers supporting their recovery by caring for their children. Research on OUD has found that familial support is beneficial in recovery, and cohesive family dynamics are associated with earlier treatment-seeking and overall lower levels of illicit drug use [36]. The participants in our study who had family support discussed its importance to their recovery. The bulk of research on the role of social and family support in treatment outcomes strongly supports improved outcomes among individuals with strong networks.

The bulk of research on religion and faith supports the idea that religious beliefs and practices are associated with recovery from substance abuse. One study found that 73% of substance abuse recovery programs in the U.S. are faith- or spirituality-based, which emphasize faith in God or a higher power [37]. This study also examined the differences in religiosity and coping among men and women and found that, over time, women showed more forgiveness of themselves and others and less negative religious coping than men [37]. This theme was supported by our research. While the fathers in our study talked about outside sources of support from family or their children’s mothers, the mothers we interviewed did not have the same level of support from their children’s fathers, but they had their faith and they had positive religious coping.

## 5. Conclusions

We used qualitative methods to explore the barriers to treatment associated with having minor children among parents enrolled in a community-based treatment program for adults in a large metropolitan area. We found significant differences in perceived barriers related to gender. Mothers were more likely to have children in their custody and perceive their children as both a barrier and a motivator for seeking treatment. Mothers were less likely to have family and social support than fathers and were more likely to feel stigma associated with drug use. Additionally, several mothers in our study identified as an ethnic minority and/or LGBTQ+ and talked about the additional stigma associated with being an ethnic or sexual minority. This study highlights the stigma faced by parents with OUD and highlights the internal and external shame felt by mothers. In addition to the suggestions from the previous literature for family-centered substance use treatment, more needs to be done to reduce the stigma and shame through the education of families, providers, and the community. The findings from this study are novel because they highlight the differences in resources and barriers that are experienced by mothers.

There are limitations to this study. This was based upon a small sample recruited from one treatment program in a large metropolitan area and may not be generalizable to a larger population. There were more mothers than fathers in our study, and participants self-selected to speak with interviewers; thus, we cannot accurately represent the views of all fathers in treatment, and self-selection bias is a factor. Owing to the supportive nature of the interviews being conducted within a treatment program, our participants may have felt more willing to share intimate details they would not have shared with outside researchers. We feel that this is a strength of the study, as are the vulnerable feelings that emerged regarding stigma.

## Figures and Tables

**Table 1:** Demographics of the parents interviewed.

ID	Gender/Sexuality	Current Age	Race/Ethnicity	Relationship Status ^1^	Ages of Children ^1^ (in Years)	Custody ^1^
F1	F—cisgender Bisexual	35–39	Black	Partnered (F/F)	4, 10	Yes
F2	F—cisgender Straight	35–39	White	Married	Pregnant, 2, 7, 10	No
F3	F—cisgender Straight	35–39	Hispanic/Latino	Single	<9, 9, 16	Yes—some of the children
F4	F—cisgender Straight	40–44	White	Divorced	9, 16	Yes
F5	F—cisgender Straight	50–54	White	Partnered	14, 18	Yes
F6	F—cisgender Straight	35–39	White	Single	6	Yes
F7	F—cisgender Gay	25–34	Hispanic/Latino	Partnered	8, 4	Yes
F8	F—cisgender Straight	45–49	Hispanic/Latino	Single	Unknown—5 children in total	Yes
F9	F—cisgender Straight	45–49	Hispanic/Latino	Married	<1, 2, 3, 6	Yes
F10	F—cisgender Gay	35–39	White	Single	11, 20	No
F11	F—cisgender Straight	45–49	Black	Single	11	Yes
M1	M—cisgender Straight	25–29	White	Single	~9	No
M2	M—cisgender Straight	25–29	White	Engaged	~10	No
M3	M—cisgender Straight	35–39	Hispanic/Latino	Single	10, 13	No

^1^ At the time of treatment; M, male; F, female.

## Data Availability

Data will be made available on request due to privacy restrictions.

## Supplementary Materials

The following supporting information can be downloaded at: https://www.mdpi.com/article/10.3390/ijerph192416660/s1, S1: Interview Guide.

## References

[1] Lipari R.N., Van Horn S.L. (2013). Children Living with Parents Who Have a Substance Use Disorder.

[2] Crowley D.M., Connell C.M., Jones D., Donovan M.W. (2019). Considering the child welfare system burden from opioid misuse: Research priorities for estimating public costs. Am. J. Manag. Care.

[3] Vanderminden J., Hamby S., David-Ferdon C., Kacha-Ochana A., Merrick M., Simon T.R., Finkelhor D., Turner H. (2019). Rates of neglect in a national sample: Child and family characteristics and psychological impact. Child Abuse Negl..

[4] Griesler P.C., Hu M.-C., Wall M.M., Kandel D.B. (2019). Nonmedical Prescription Opioid Use by Parents and Adolescents in the US. Pediatrics.

[5] Feder K.A., Mojtabai R., Musci R.J., Letourneau E.J. (2018). U.S. adults with opioid use disorder living with children: Treatment use and barriers to care. J. Subst. Abus. Treat..

[6] Brady T.M., Silber A.O. (2005). Women in Substance Abuse Treatment: Results from the Alcohol and Drug Services Study (ADSS).

[7] Greenfield S.F., Grella C.E. (2009). What is “women-focused” treatment for substance use disorders?. Psychiatr. Serv..

[8] Krans E.E., Patrick S.W. (2016). Opioid Use Disorder in Pregnancy: Health Policy and Practice in the Midst of an Epidemic. Obstet. Gynecol..

[9] National Institute on Drug Abuse Overdose Death Rates. https://www.drugabuse.gov/related-topics/trends-statistics/overdose-death-rates.

[10] Koons A.L., Rayl Greenberg M., Cannon R.D., Beauchamp G.A. (2018). Women and the Experience of Pain and Opioid Use Disorder: A Literature-based Commentary. Clin. Ther..

[11] Leone B., Di Nicola M., Moccia L., Pettorruso M., De Risio L., Nucara G., Zamboni L., Callea A., Janiri L., Cibin M. (2017). Gender-related psychopathology in opioid use disorder: Results from a representative sample of Italian addiction services. Addict. Behav..

[12] Huhn A.S., Tompkins D.A., Campbell C.M., Dunn K.E. (2019). Individuals with Chronic Pain Who Misuse Prescription Opioids Report Sex-Based Differences in Pain and Opioid Withdrawal. Pain Med..

[13] Huhn A.S., Dunn K.E. (2020). Challenges for Women Entering Treatment for Opioid Use Disorder. Curr. Psychiatry Rep..

[14] Unger A., Jung E., Winklbaur B., Fischer G. (2010). Gender issues in the pharmacotherapy of opioid-addicted women: Buprenorphine. J. Addict. Dis..

[15] Rhee T.G., Peltier M.R., Sofuoglu M., Rosenheck R.A. (2020). Do Sex Differences Among Adults with Opioid Use Disorder Reflect Sex-specific Vulnerabilities? A Study of Behavioral Health Comorbidities, Pain, and Quality of Life. J. Addict. Med..

[16] Gannon M., Short V., LaNoue M., Abatemarco D. (2021). Prevalence of Adverse Childhood Experiences of Parenting Women in Drug Treatment for Opioid Use Disorder. Community Ment. Health J..

[17] Bawor M., Dennis B.B., Varenbut M., Daiter J., Marsh D.C., Plater C., Worster A., Steiner M., Anglin R., Pare G. (2015). Sex differences in substance use, health, and social functioning among opioid users receiving methadone treatment: A multicenter cohort study. Biol. Sex Differ..

[18] Langabeer J., Champagne-Langabeer T., Luber S.D., Prater S.J., Stotts A., Kirages K., Yatsco A., Chambers K.A. (2020). Outreach to people who survive opioid overdose: Linkage and retention in treatment. J. Subst. Abuse Treat..

[19] Malterud K. (2001). Qualitative Research: Standards, Challenges, and Guidelines. Lancet.

[20] Taplin S., Mattick R.P. (2015). The nature and extent of child protection involvement among heroin-using mothers in treatment: High rates of reports, removals at birth and children in care. Drug Alcohol Rev..

[21] Elms N., Link K., Newman A., Brogly S.B. (2018). Need for women-centered treatment for substance use disorders: Results from focus group discussions. Harm Reduct. J..

[22] McMahon T.J., Winkel J.D., Suchman N.E., Luthar S.S. (2002). Drug dependence, parenting responsibilities, and treatment history: Why doesn’t mom go for help?. Drug Alcohol Depend..

[23] McMahon T.J., Winkel J.D., Luthar S.S., Rounsaville B.J. (2005). Looking for poppa: Parenting status of men versus women seeking drug abuse treatment. Am. J. Drug Alcohol Abus..

[24] Andrews C.M., Humphreys K. (2019). Investing in Medicaid to End the Opioid Epidemic. Psychiatr. Serv..

[25] Medicaid and CHIP Payment and Access Commission (MACPAC) (2021). Chapter 2: Advancing Maternal and Infant Health by Extending the Postpartum Coverage Period.

[26] Ranji U., Gomez I., Salganicoff A. Expanding Postpartum Medicaid Coverage. https://www.kff.org/womens-health-policy/issue-brief/expanding-postpartum-medicaid-coverage/.

[27] Brundage S.C., Levine C. (2019). The Ripple Effect: The Impact of the Opioid Epidemic on Children and Families.

[28] Burghardt L.C., Ayers J.W., Brownstein J.S., Bronstein A.C., Burns Ewald M., Bourgeois F.T. (2013). Adult Prescription Drug Use and Pediatric Medication Exposures and Poisonings. Pediatrics.

[29] Champagne-Langabeer T., Cardenas-Turanzas M., Ugalde I.T., Bakos-Block C., Stotts A.L., Cleveland L., Shoptaw S., Langabeer J.R. (2022). The Impact of Pediatric Opioid-Related Visits on U.S. Emergency Departments. Children.

[30] Crawford A.D., McGlothen-Bell K., Recto P., McGrath J.M., Scott L., Brownell E.A., Cleveland L.M. (2022). Stigmatization of Pregnant Individuals with Opioid Use Disorder. Womens Health Rep..

[31] Tsuda-McCaie F., Kotera Y. (2022). A qualitative meta-synthesis of pregnant women’s experiences of accessing and receiving treatment for opioid use disorder. Drug Alcohol. Rev..

[32] Pazol K., Ellington S.R., Fulton A.C., Zapata L.B., Boulet S.L., Rice M.E., Cox S., Romero L., Lathrop E., Hurst S. (2018). Contraceptive Use among Women at Risk for Unintended Pregnancy in the Context of Public Health Emergencies—United States, 2016. MMWR Morb. Mortal. Wkly. Rep..

[33] Knight K.R. (2020). Structural Factors That Affect Life Contexts of Pregnant People with Opioid Use Disorders: The Role of Structural Racism and the Need for Structural Competency. Women’s Reprod. Health.

[34] Harris J., McElrath K. (2012). Methadone as Social Control: Institutionalized Stigma and the Prospect of Recovery. Qual. Health Res..

[35] Grella C.E., Needell B., Shi Y., Hser Y.I. (2009). Do drug treatment services predict reunification outcomes of mothers and their children in child welfare?. J. Subst. Abuse Treat..

[36] Cooper S., Nielsen S. (2017). Stigma and Social Support in Pharmaceutical Opioid Treatment Populations: A Scoping Review. Int. J. Ment. Health Addict..

[37] Grim B.J., Grim M.E. (2019). Belief, Behavior, and Belonging: How Faith is Indispensable in Preventing and Recovering from Substance Abuse. J. Relig. Health.

