# Supplementary material for: Experiences of Parents with Opioid Use Disorder during Their Attempts to Seek Treatment: A Qualitative Analysis
Source: Int J Environ Res Public Health. 2022 Dec 11;19(24):16660. doi: 10.3390/ijerph192416660 (PMC9779200; doi:10.3390/ijerph192416660)
Supplement: Supplementary file 1 [file ijerph-19-16660-s001.zip › ijerph-2061138-supplementary.pdf]

S1. Interview guide.

The purpose of this study is to help guide professionals and other parents who struggle with OUD in supporting their children and we have two primary aims

- *To help us understand from a first-hand perspective the impact of parents' opioid addiction and subsequent recovery on children, parenting, and family systems.*
- *To discover the most helpful and least helpful sources of support parents rely on to help their children during active addiction, treatment, and recovery.*

1. Can you please tell me a little about your child(ren) and share some pictures?
2. Can you briefly recount the story of how you went from opioid use to misuse, to addiction and eventually treatment and recovery?
    - What helped or hindered your seeking and finding treatment?
3. What concerns did you have for your child(ren) during that time and how did you manage?
4. What sources of support did you rely on to help you and your children? (This could include personal, familial or community resources.)
    - the pros and cons of each for your situation
5. How have things changed since you began in recovery?
    - In your parent-child relationship?
    - For your child(ren)?
    - In your family system?
    - Your sources of support?
6. What advice would you give to parents who are currently struggling with OUD?
